# Editorial: Bacterial Transcription Factors and the Cell Cycle

**DOI:** 10.3389/fmicb.2021.821394

**Published:** 2021-12-24

**Authors:** Morigen, Monika Glinkowska, Jianping Xie

**Affiliations:** ^1^State Key Laboratory of Reproductive Regulation and Breeding of Grassland Livestock, School of Life Sciences, Inner Mongolia University, Hohhot, China; ^2^Department of Bacterial Molecular Genetics, University of Gdansk, Gdańsk, Poland; ^3^Institute of Modern Biopharmaceuticals, School of Life Sciences, Southwest University, Chongqing, China

**Keywords:** bacterial transcription factors, chromosome replication, cell division, cell growth, cell cycle

## Introduction

The fluctuation in gene expression regulated by transcription factors are tightly associated with progression of the bacterial cell cycle. In fact, the replication initiator protein DnaA regulates DNA replication (Fuller and Kornberg, 1983; Bramhill and Kornberg, 1988) and also controls gene expression as a transcription factor (Atlung et al., 1984; Braun et al., 1985; Messer and Weigel, 1997; Wurihan et al., 2018). It is likely that DnaA may coordinate DNA replication and repair process by participating in control of the SOS response genes (Wurihan et al., 2018). As reviewed by Menikpurage et al., DnaA has been shown to regulate expression of genes involved in different cellular processes including quorum sensing, cell motility, heat shock response, DNA repair, and cell cycle regulation. In turn, the quorum sensing regulators QseB and QseC in *Escherichia coli* have been reported to modulate the expression of *dnaA* (Wu et al., 2021). Thus, dual function DnaA might temporally coordinate diverse cellular events with the onset of chromosome replication. Especially, in *Caulobacter crescentus*, DnaA couples DNA replication with the expression of the two oscillating regulators GcrA and CtrA, and the DnaA/GcrA/CtrA regulatory cascade drives forward the progression of the cell cycle (Collier et al., 2006). Other transcription factors and proteins involved in transcription control have also been shown to coordinate the cell cycle events. Namely, RpoS in *Coxiella* regulates the gene expression involved in the developmental cycle (Moormeier et al., 2019). Similarly, BolA in most Gram-negative bacteria turns off motility and turns on biofilm development as a transcription factor (Dressaire et al., 2015), whereas CtrA regulates cell division and outer membrane composition of the pathogen *Brucella abortus* (Francis et al., 2017). The Research Topic is a collection of articles focusing on the *Bacterial Transcription Factors and the Cell Cycle*, particularly: initiation of chromosome replication, cell division, and cell growth.

## The Bacterial Transcription Factors

Mfd is a transcription-coupled DNA repair factor, which couples a stalled RNA polymerase with the nucleotide excision repair pathway to preferentially repair lesions in the template strand of actively transcribing genes (Hanawalt and Spivak, 2008). Interestingly, Martin et al. find that Mfd modulates global transcription in stationary-phase and during disulfide stress in *Bacillus subtilis* cells. Loss of Mfd leads to changes in expression of genes involved in translation, endospore formation, motility, and pyrimidine metabolism. The profound effects of Mfd on the modulation of the transcriptome and on bacterial physiology, particularly in cells experiencing nutritional and oxidative stress suggest that Mfd is multifunctional protein coordinating transcription and DNA repair.

Regulation of gene expression by various transcription factors enables bacteria to adapt to various environmental conditions and utilize different nutrients. In this context, transcription factors and their functions are investigated in various bacterial species in the collection. Firstly, Tsevelkhoroloo et al. elucidates DagR-mediated regulation of expression of three agarase genes (*dagA, dagB*, and *dagC* in *Streptomyces coelicolor*). They show that DagR operates by binding to a palindromic sequence present in the upstream regions of the three genes. This is the first report on the regulation of expression of genes involved in agar metabolism in *S. coelicolor* A3(2). In *Mycobacterium smegmatis*, Jeong-Il Oh lab demonstrates the distinct roles of additional RsbW homologs: RsbW1, RsbW2, and RsbW3 in the SigF Partner Switching System (PSS). They show that RsbW1 and RsbW2 serve as the anti-sigma factor of SigF and the protein kinase phosphorylating RsfB, respectively, while RsbW3 functions as an anti-SigF antagonist through its protein interaction with RsbW1. Moreover, they also demonstrate that Crp1 plays a predominant role in induction of the cydAB operon in *M. smegmatis* exposed to the respiratory electron transport chain (ETC)-inhibitory conditions. In a similar context, Wan et al. identify that OxyR as a master transcription regulator mediates cellular responses to hydrogen peroxide in *Yersinia pseudotuberculosis*. Using genomic and transcriptomic analyses, they show that OxyR activates transcription of diverse genes encoding for catalases, peroxidases, and thiol reductases. Those findings provide not only novel insights into the structural and functional diversity of bacterial hydrogen peroxide-scavenging systems but also a basic understanding of how *Y. pseudotuberculosis* copes with oxidative stress. Finally, in *Bacillus anthracis*, the NprR is a protein that exhibits moonlighting functions as either a phosphatase or a neutral protease regulator that belongs to the RNPP family. Wang et al. show that the extracellular protease activity of an *nprR* deletion mutant is significantly decreased. Further, the data suggests that NprR is a transcription regulator that controls genes involved in extracellular proteolysis and also the oxidative stress response.

## The Bacterial Cell Cycle

The fast-growing *E. coli* cell cycle consists of a chromosome replication and a cell division cycle (Boye and Nordström, 2003). As reviewed by Grimwade and Leonard, chromosome replication is a critical event in the cell cycle since all daughter cells must inherit a complete genome. In bacteria, chromosome replication starts when nucleoprotein complexes (orisomes) unwind replication origin DNA and build new replication forks. Functional orisomes comprising DnaA bound to a set of high and low affinity recognition sites in *oriC* must be assembled once, and only once, per cell cycle. During the orisome assembly in *E. coli*, three known transcriptional factors, SeqA, Fis (factor for inversion stimulation), and IHF (integration host factor), interact directly with the *oriC* site to regulate orisome assembly and replication initiation timing. Interestingly, Kasho et al. determine the *E. coli* genome-wide, cell cycle-dependent binding of IHF with base-pair resolution using GeF-seq (genome footprinting with high-throughput sequencing). They show that *oriC* is a main site for stable IHF binding before replication initiation whereas all other loci have a reduced IHF binding. Thus, they believe that IHF rapidly dissociates from *oriC* after replication initiation, and IHF binding to other sites is sustained or stimulated. The findings provide mechanistic insight into cell cycle-dependent IHF binding and dissociation in the genome. In another work concerning nucleoid associated proteins, Pal et al. characterize the YbaB/EbfC family DNA binding protein *of C. crescentus*, which is conserved in bacteria. Unlike *Borrelia burgdorferi* EbfC protein, YbaBCc protein is a non-specific DNA-binding protein that protects DNA from enzymatic degradation. The findings reveal that YbaBCc is a small histone-like protein and may play a role in bacterial chromosome structuring and gene regulation.

Cell division is tightly coordinated with chromosome replication, and cell growth. In *E. coli*, many cell division proteins interact with FtsZ and modulate Z-ring assembly, cell wall insertion and peptidoglycan remodeling. DiBiasio et al. show that ZapE directly interacts with FtsZ and phospholipids *in vitro*. ZapE is an ATPase that accumulates during late constriction, and induces bundling of GTP-induced FtsZ polymers in the presence of ATP. ZapE is proposed to ensure cell proliferation during and after stress, especially when cells are also deleted for *minC*. This suggests that ZapE is a stress-regulated cell division protein, promoting growth and division after exposure to environmental stress. In another study, Patil et al. show that excess DnaA(L366K) or deletion of *fis* allows for growth of cells that otherwise would undergo cell cycle arrest as a result of accumulation of lipoprotein Lpp(C21G). The DnaA(L366K)-mediated restoration of growth occurs by reduced expression of Lpp(C21G) via a σE-dependent small-regulatory RNA (sRNA), MicL-S. On the other hand, deletion of *fis* rescues Lpp(C21G)-evoked growth arrest in cells lacking physiological levels of PG and cardiolipin (CL), independently of MicL-S. The results suggest a close link between the physiological state of the bacterial cell membrane and DnaA- and Fis-dependent growth.

Quantitative models of the cell-cycle and cell-size control in *E. coli* and *B. subtilis* have been proposed over the last decade to explain single-cell experimental data generated with high-throughput methods. Le Treut et al. examine predictive power of these models against experimental data. They find that Independent Double Adder (IDA) model is more consistent with the data than the other models and conclude that the IDA model is so far the most consistent one with both the data and the biology of bacterial cell-cycle and cell-size control.

## Perspectives

The location of genes relative to *ori* and *ter* of the bacterial circular chromosome has profound effects on transcription regulatory networks and physiological processes (Sobetzko et al., 2012). The CtrA phosphorelay is conserved in most alphaproteobacteria as a gene regulatory system. Tomasch et al. show that the CtrA phosphorelay genes are conserved closer to *ori* in the Caulobacterales, while the genes are highly conserved closer to *ter* in the Rhodobacterales. However, the location of the phosphorelay genes is the least conserved in the Rhodospirillales and Sphingomonadales. The findings suggest that selection pressure results in differential positioning of CtrA phosphorelay and associated genes in alphaproteobacteria. Thus, future deeper investigations on correlation of chromosomal locations and function of a broad spectrum of bacterial transcription factors are worth pursuing both theoretically and experimentally.

## Author Contributions

M, MG, and JX participated in the first manuscript draft. M edited the final version and all authors revised it. All authors listed have made a substantial, direct, and intellectual contribution to the work and approved it for publication.

## Funding

This work was supported by a grants from the National Natural Science Foundation of China NSFC (Grant no. 32060016 to M).

## Conflict of Interest

The authors declare that the research was conducted in the absence of any commercial or financial relationships that could be construed as a potential conflict of interest.

## Publisher's Note

All claims expressed in this article are solely those of the authors and do not necessarily represent those of their affiliated organizations, or those of the publisher, the editors and the reviewers. Any product that may be evaluated in this article, or claim that may be made by its manufacturer, is not guaranteed or endorsed by the publisher.
